# Relationship between the Charlson Comorbidity Index and cost of treating hip fractures: implications for bundled payment

**DOI:** 10.1007/s10195-015-0337-z

**Published:** 2015-02-20

**Authors:** Daniel J. Johnson, Sarah E. Greenberg, Vasanth Sathiyakumar, Rachel Thakore, Jesse M. Ehrenfeld, William T. Obremskey, Manish K. Sethi

**Affiliations:** The Vanderbilt Orthopaedic Institute Center for Health Policy, Vanderbilt University, Suite 4200, South Tower, MCE, Nashville, TN 37221 USA

**Keywords:** Charlson Comorbidity Index, Costs, Length of stay (LOS)

## Abstract

**Background:**

The aim of this study is to investigate how the Charlson Comorbidity Index (CCI) scores contribute to increased length of stay (LOS) and healthcare costs in hip fracture patients.

**Materials and methods:**

Through retrospective analysis at an Urban level I trauma center, charts for all patients over the age of 60 years who presented with low-energy hip fracture were evaluated. 615 patients who underwent operative fixation of hip fracture or hemiarthroplasty secondary to hip fracture were identified using Current Procedural Terminology (CPT) codes search and included in the study. Data was collected on patient demographics, medical comorbidities, and hospitalization length; from this, the CCI score and the cost to the institution (with an average cost/day of inpatient stay of $4,530) were calculated.

**Results:**

Multivariate linear regression analysis modeled the length of stay as a function of CCI score. Each unit increase in the CCI score corresponded to an increase in length of hospital stay and hospital costs incurred [effect size = 0.21; (0.0434–0.381); *p* = 0.014]. Patients with a CCI score of 2 (compared to a baseline CCI score of 0), on average, stayed 1.92 extra days in the hospital, and incurred $8,697.60 extra costs.

**Conclusions:**

The CCI score is associated with length of stay and hospital costs incurred following treatment for hip fracture. The CCI score may be a useful tool for risk assessment in bundled payment plans.

**Level of evidence:**

Level III.

## Introduction

Hip fracture procedure volumes have risen in recent years, largely due to an aging population, and this trend is expected to increase dramatically in the coming decades, from 250,000 procedures annually to 500,000 by 2040 [1]. With current estimates of treating a hip fracture averaging $11,844–13,805, bundled payments have been proposed to contain costs without sacrificing quality in hip fracture treatment [2–4]. Bundled payments, otherwise known as episode-of-care payments, set a fixed reimbursement amount that collectively holds all providers responsible for patient outcomes. A key component of episode-based payment is that it attributes an episode of care as the length of time that an “average” patient would need for a certain intervention, and any increase in cost due to an unplanned prolonged length of stay (LOS) may have a significant negative financial impact on any institution caring for a hip fracture patient [5]. To protect the institution from incurring such costs, it is imperative to identify the patient factors that are associated with increased costs, and to develop methods to standardize their weighting and quantify their economic impact.

A number of scoring systems which summarize the patient’s overall health status have been developed, including the American Society of Anesthesiologist’s score (the ASA score), the Elixhauser score, and the Charlson Comorbidity Index (CCI). Higher ASA scores have been shown to be associated with increased hospital costs secondary to increased LOS in hip fracture patients [6]. Similarly, work by Nikkel et al. [2] demonstrated that higher Elixhauser scores are correlated with increased length of hospitalization and hospital costs incurred in hip fracture patients. Higher Charlson Comorbidity Index scores have been shown to correlate with increased 30-day mortality after hip fractures [7], increased 90-day mortality after hip fractures [8], increased in-hospital mortality in patients with hip fractures [9], and readmission rates after orthopedic procedures, including treatment of hip fractures [10]. Data about the relationship between CCI and LOS following hip fracture is limited, and at the present time, there are no studies to our knowledge, looking at the relationship between CCI scores and length of hospitalization in the United States; therefore, this study assesses the relationship between CCI, as a useful indicator of patient health and LOS following hip fracture, and estimates additional hospital costs that may be used to weight bundled payments.

## Materials and methods

Institutional review board approval was obtained for this study. This was a retrospective cohort study that included all patients who underwent operative fixation of hip fracture or hemiarthroplasty secondary to hip fracture, including both femoral neck fractures and intertrochanteric fractures, at Vanderbilt University Medical Center, a level one trauma center, from January 2000 to December 2009. Current Procedural Terminology (CPT) codes were used to find patients who had experienced a hip fracture from a low-energy fall and received an intervention of cephalomeduallary nailing (CMN), closed reduction and percutaneous pinning (CRPP), total hip arthroplasty (THR), hemiarthroplasty (hemi), or open reduction internal fixation (ORIF). All patients over the age of 60 years with acetabular, proximal femoral, femoral neck, and trochanteric fractures were selected. Patients with incomplete medical records were excluded. Additional demographic and clinical covariates were collected from our institution’s electronic medical records database. Medical comorbidities were documented preoperatively by routine preoperative assessment, and, from this data, the Charlson Comorbidity Index was calculated according to Deyo’s description [11].

The average total cost to the hospital of an inpatient day ($4,530 per day) was obtained from the institution’s financial services and the average cost was treated as a unit cost per inpatient day. All fractional LOS values were rounded to the nearest whole number and multiplied by the per day cost.

The primary outcome of interest was the relationship between the CCI and the length of hospitalization. Risk of the occurrence of the outcome of interest (i.e. LOS) was modeled as a function of the preoperative CCI using multivariable linear regression. The multivariate linear regression model controlled for confounders (gender, ASA, body mass index, race, smoking status, anesthesia type and comorbidities) previously found to be associated with the outcome (i.e. prolonged LOS). Statistical significance was set at *p* = 0.05.

## Results

Six hundred and fifteen complete records were obtained for isolated low-energy hip fractures in patients 60 years or older who were treated at our Level 1 trauma center. The average age of the hip fracture patient was 78.4 years and 51.7 % of our patients were aged 75–89 years. Caucasians comprised the majority of our patient cohort (84.7 %), followed by African-Americans (7.3 %). Nearly three-quarters of our patient cohort had a CCI score less than 3, and more than half of the cohort had a CCI score of either 0 or 1. Patient characteristics and demographic data are summarized in Table 1.

**Table 1:** Demographic information

	*N*	%
Age (years)		
60–64	76	12.4
65–69	67	10.9
70–74	77	12.5
75–79	107	17.4
80–84	106	17.2
85–89	105	17.1
>90	77	12.5
Gender		
Male	201	32.7
Female	414	67.3
Race		
African-American	45	7.3
Asian	3	0.5
Caucasian	521	84.7
Hispanic/Latino	2	0.3
Declined to volunteer	44	7.2
Current smoker		
No	599	97.4
Yes	16	2.6
CCI Score		
0	179	29.1
1	165	26.8
2	110	17.9
3	58	9.4
4	37	6.0
5	13	2.1
6	13	2.1
7	11	1.8
8	11	1.8
9	10	1.6
10	3	0.5
11	3	0.5
12	1	0.2
13	0	0.0
14	1	0.2

The different surgical procedures performed, classified by CPT codes, and the average LOS and hospital costs incurred for the inpatient stay are summarized in Table 2. The three most common procedures, representing 52.7 % of the procedures performed, were partial hip hemiarthroplasty (CPT code 7125; 19.7 %), open reduction and internal fixation of inter/per/subtrochanteric fracture with plate or screw, with/without cerclage (CPT 27244; 19.0 %), and open reduction and internal fixation of femoral neck fracture (CPT 27236; 14.0 %). These three procedures had an average LOS of 7.37 days with an average cost of $33,401. Overall, for all the procedures, the average LOS was 5.84 days and the average cost was $26,470 with a median of $27,180.

**Table 2:** Procedures

CPT code	Procedure	Number of cases	Percentage (%)	Average LOS (days)	Average cost ($4530 per day)
75.35	Insertion of intramedullary nail – femur	6	1.0	6.00	$27,180.00
78.59	Percutaneous pinning of hip	14	2.3	5.36	$24,280.80
79.352	Open reduction internal fixation of femoral neck	1	0.2	3.00	$13,590.00
79.353	Open reduction internal fixation of femoral head	2	0.3	3.00	$13,590.00
79.783	Percutaneous pinning of lower extremity	2	0.3	5.50	$24,915.00
79.855	Open reduction internal fixation of hip with compression screw and plate	3	0.5	6.00	$27,180.00
79.857	Open reduction internal fixation of intertrochanteric fx	20	3.3	5.05	$22,876.50
81.6	Arthroplasty of hip – total primary	2	0.3	6.50	$29,445.00
846	Hemiarthroplasty of hip	29	4.7	5.95	$26,953.50
7125	Hemiarthroplasty hip – partial	121	19.7	7.72	$34,971.60
27130	Arthroplasty, acetabular and proximal femoral prosthetic replacement with or without autograft/allograft	37	6.0	8.41	$38,097.30
27130A	Arthroplasty, acetabular and proximal femoral prosthetic replacement with or without autograft/allograft, anterior	5	0.8	9.00	$40,770.00
27235	Percutaneous skeletal fixation, femoral fx, proximal, neck	45	7.3	5.67	$25,685.10
27236	Treatment, open femoral fx, proximal end, neck, internal fixation/prosthetic replacement	86	14.0	7.12	$32,253.60
27244	Treatment, inter/per/subtrochanteric femoral fx, with plate/screw type implant,with or without cerclage	117	19.0	7.28	$32,978.40
27245	Open treatment, inter/per/subtrochanteric femoral fx, with intermedullary implant, with or without screw/cerclage	82	13.3	6.95	$31,483.50
27248	Open treatment, greater trochanteric fx, with or without internal or external fixation	5	0.8	4.40	$19,932.00
27254	Open treatment, hip dislocation, traumatic, with acetabular wall/femoral head fx, with or without internal or external fixation	1	0.2	7.00	$31,710.00
27506	Open treatment, femoral shaft fx, with insertion, intramedullary implant, with or without screw/cerclage	30	4.9	6.30	$28,539.00
27507	Open treatment, femoral shaft fx, with plate/screws, with or without cerclage	6	1.0	3.50	$15,855.00
27509	Percutaneous skeletal fixation, femoral fx, distal end	1	0.2	3.00	$13,590.00

Male gender (which represented 33 % of our cohort) was also significantly associated with an additional 1.12 (95 % CI 0.375–1.865) days in hospital (*p* = 0.003); the financial implication of this finding is that each male patient costs the hospital an additional $5,073.60 as compared to a female patient (see Table 1). There was also an association between smoking status and hip fractures, but this did not reach statistical significance, probably due to the lack of power as there were only 16 current smokers, representing 2.6 % of our patient cohort.

**Fig. 1:**
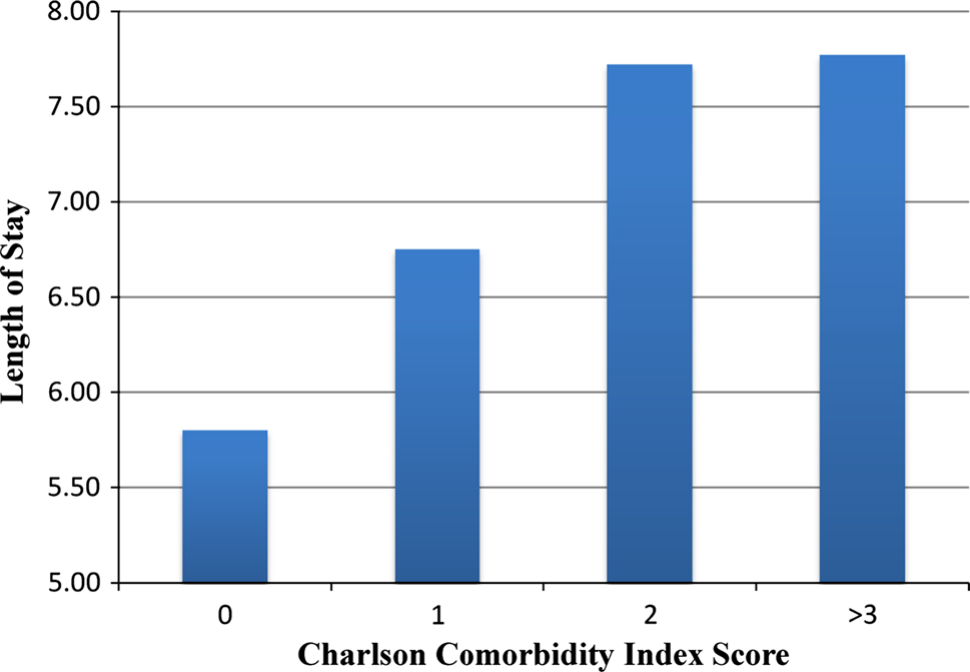
Mean length of stay per CCI score calculated from patient’s medical comorbidities found in charts

**Fig. 2:**
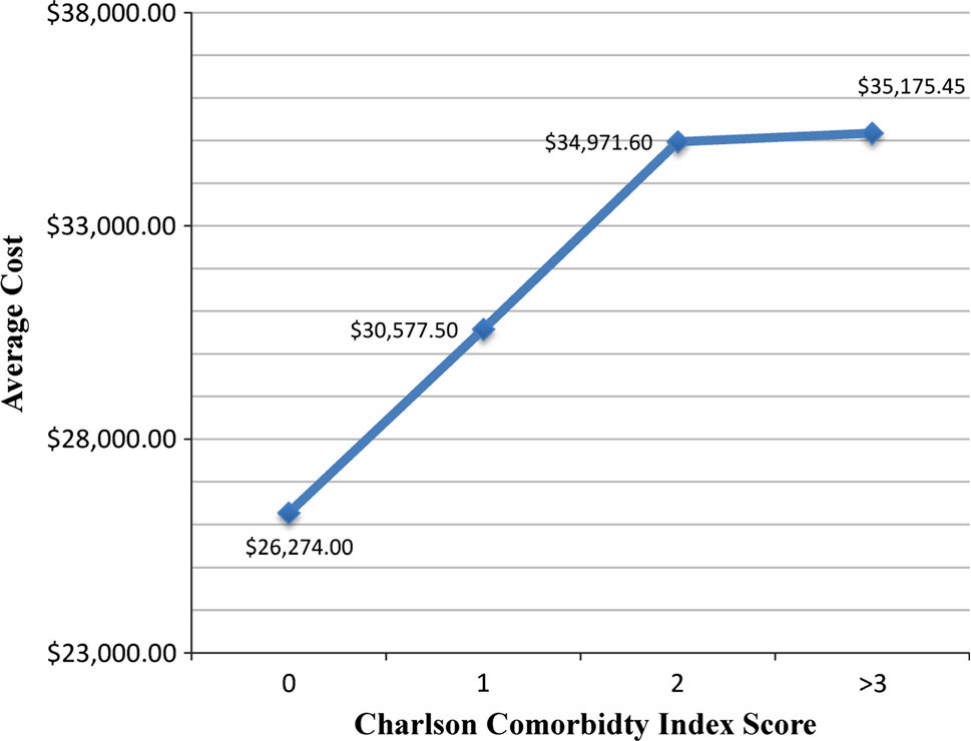
Mean cost of stay per CCI score

There was an association between CCI score and LOS [effect size: 0.21 (0.0434–0.381); *p* = 0.014] with higher CCI scores having an increased likelihood of longer hospital LOS, and consequently higher costs, as summarized in Figs. 1 and 2. The average LOS for our patients with a CCI score of 0 was 5.8 days ($26,274.00); patients with a CCI score of 1 had an average LOS of 6.5 days ($30,577.50); patients with a CCI score of 2 had an average LOS of 7.72 days ($34,971.60); patients with a CCI score of three or greater had an average LOS of 7.77 days ($35,175.45). Therefore, the financial difference between treating a patient with a CCI score of 0 as compared to a patient with a CCI score of 2 was an additional $8,697.60 per patient.

## Discussion

We found that increasing CCI scores are associated with longer LOS following hip fracture, and we quantified the cost burden attributable to this prolonged LOS. Our finding supports the work of other authors who have noted a relationship between comorbidities and prolonged LOS and increased hospital costs following hip fracture [2]; however, our study is the first to assess this relationship using the CCI.

Because there are currently no other published studies examining the relationship between the CCI score and LOS and hospital costs following hip fractures, we compared our findings with those reported for total joint arthroplasty. In the Tien et al. [12] study of total joint arthroplasty in Taiwan, a CCI score of 1 or higher correlated well with length of hospitalization and higher hospital costs. In parallel, our study is the first to suggest that an increased CCI score is associated with a prolonged LOS and increased hospital costs after hip fracture treatment. The relationship between the CCI score and LOS following different procedures implies that the CCI score can succinctly summarize a patient’s overall health status, and therefore makes it a versatile tool to use for risk stratification in negotiating bundled payments. The CCI score has also been shown to be associated with short-term mortality following hip fractures [7–9] and a relationship between higher CCI scores and readmission rates following any orthopedic procedure has also been identified [10].

There are several factors to consider in the interpretation of our results. First, the comorbidities of our patient population and the cost of inpatient care reflect the practice of a single, tertiary care, academic medical center, and further analysis is necessary to determine whether our findings are applicable to other surgical settings. Secondly, we only evaluated bundled payments that were related to the inpatient cost from the index procedure, and although this limitation does not affect our findings regarding the association between the CCI score and increased hospital costs, it is important to recognize that the cost burden we found represents the minimum additional cost incurred, and more research is needed to quantify the relationship between the CCI score and other factors which would affect hospital costs in a bundled payment model.

The rationale for bundled payments is to incentivize various providers to collaboratively deliver high-quality care at the lowest possible cost, but several authors have noted potential downsides of this payment model, both for the patients and the providers. With respect to the former, Bozic et al. [13] noted that bundled payment models simultaneously create the incentive to withhold care, and because of this, there is a growing awareness that institutions need methods to calculate the specific cost burden of patient factors associated with a particular procedure prior to entering into a bundled payment reimbursement agreement [5]. This is not only imperative for the financial solvency of the institution [5], but it is also necessary to ensure that more complex patients with multiple comorbid conditions receive the care they need. The results of our study, which show the impact of increasing CCI scores on hospital LOS and its financial implications further highlight the importance of quantifying the specific cost burden of patient factors, both to protect the financial interests of the institution and to ensure that funds are allotted to meet the needs of medically complex patients. In summary, the results of our study suggest that the CCI score may have a role in predicting hospital costs and negotiating reimbursement rates for the treatment of hip fractures, and, based on our results, more research is warranted to evaluate the impact of the CCI score on other costs included in bundled payments.
